# IFN-γ Triggered STAT1-PKB/AKT Signalling Pathway Influences the Function of Alloantigen Reactive Regulatory T Cells

**DOI:** 10.1111/j.1600-6143.2009.02858.x

**Published:** 2010-01

**Authors:** B Wei, S Baker, J Wieckiewicz, K J Wood

**Affiliations:** a Transplantation Research Immunology Group, Nuffield Department of Surgery, John Radcliffe Hospital, University of OxfordOxford OX3 9DU, UK; b Laboratory of Molecular Cell Biology, Institute of Biochemistry and Cell Biology, Chinese Academy of SciencesShanghai 200031, China (current address)

**Keywords:** Akt survival pathway, allograft survival, interferon gamma (IFN-γ), regulatory T cells, STAT-1

## Abstract

CD4^+^CD25^+^Foxp3^+^ regulatory T cells (Tregs) play a key role in the induction and maintenance of peripheral tolerance. Rapid and transient production of IFN-γ by Tregs from mice tolerized to alloantigen *in vivo* has been shown to be critical for their regulatory function. This IFN-γ has the potential to affect the function of cells present in the same local microenvironment as the Tregs, including the Tregs themselves. Here we investigated the mechanism by which IFN-γ produced by Tregs triggered signaling pathways in alloantigen reactive Tregs themselves thereby influencing their function *in vivo*. We show that IFN-γ production and STAT1 activation was increased, while STAT1-dependent PKB/AKT activation was downregulated in alloantigen reactive Tregs. Further, the activation of STAT1 was blocked in IFN-γ receptor deficient as well as IFN-γ–deficient Tregs, suggesting that IFN-γ produced by the alloantigen reactive Tregs might act in an autocrine manner to induce STAT1 activation. Importantly, STAT1-deficient Tregs failed to control allograft rejection *in vivo*. Overall, these findings suggest that the IFN-γ–induced STAT1-PKB/AKT signaling pathway plays a key role in upregulating the ability of alloantigen reactive Tregs to control graft rejection *in vivo.*

## Introduction

CD4^+^CD25^+^Foxp3^+^ regulatory T lymphocytes (Tregs) are specialized T cells that play a critical role in the control of immune homeostasis, the maintenance of immunological self-tolerance and in the regulation of antigen-induced responses including tumor immunity and transplantation (1). A key role for IFN-γ in tolerance induction has been demonstrated in allergy, cell and organ transplantation, and autoimmunity including experimental autoimmune allergic encephalomyelitis (EAE) and glomerulonephritis (2–4). It has been reported that a significant amount of IFN-γ is produced by PMA-/ionomycin-stimulated Foxp3^+^ Tregs *in vitro* (5), and that Tregs are unable to prevent collagen induced arthritis in IFN-γ receptor deficient (IFN-γR^−/−^) mice (6). In addition, alloantigen reactive CD4^+^CD25^+^ Tregs from tolerized mice, which were induced *in vivo* by pretreatment with donor alloantigen under the cover of anti-CD4 therapy, upregulate IFN-γ expression rapidly and transiently with enhanced regulatory activity (7). However, it remains unclear how IFN-γ produced by Tregs themselves might influence their suppressive function *in vivo*.

IFN-γ has been identified as the activator of the JAK-STAT1 signal transduction pathway in various cell types through its specific receptor, the IFN-γR (8). Binding of IFN-γ to IFN-γR induces IFN-γR oligomerization and activation of JAK1 and JAK2 by *trans*-phosphorylation. The activated JAKs phosphorylate the intracellular domain of the IFN-γR that then serves as a docking site for STAT1. STAT1 is phosphorylated on tyrosine 701, undergoes dimerization and translocates to the nucleus to promote IFN-γ–regulated gene expression (9). It has been reported that STAT1-deficient mice have reduced numbers of naturally occurring CD4^+^CD25^+^ Tregs and develop EAE with increased frequency (10). Despite these observations, whether IFN-γ triggered STAT1 activation plays an important role in regulating the suppressive function of Tregs *in vivo* is not clear.

In addition, it has been reported that biological response to IFN-γ is also pleiotropic since the cytokine engages multiple signal-transduction pathways, including STAT1 dependent PI3’K-PKB/AKT, MEK-ERK or STAT1 independent pathways (9). Previous studies have reported that mouse CD4^+^CD25^+^ T cells reduce activation of PKB/AKT following TCR/CD28 stimulation (11) and expression of the active form of AKT suppresses the function of CD4^+^CD25^+^ Tregs without affecting Foxp3 expression (12). However, this remains controversial since others showed that continued TCR signaling and constitutive PI3K/AKT/mTOR activity regulated Foxp3 expression in CD4^+^Foxp3^+^ cells (13). In addition, IL-2 plays an essential role in the development of CD4^+^CD25^+^ T cells during the neonatal period as well as in the maintenance of the Treg population *in vivo* (14). It has been suggested that IL-2 regulates Foxp3 expression in CD4^+^CD25^+^ T cells through a STAT5-dependent mechanism (15). Therefore, we were interested in whether AKT, ERK or STAT5 activity is linked to the IFN-γ–STAT1 pathway and enhances the suppressive function of alloantigen reactive Tregs.

In this study, we show that the phosphorylation level of STAT1, but not of STAT5 or ERK1/2, was specifically increased in Tregs from mice tolerized to alloantigen that also upregulated production of IFN-γ. By contrast, STAT1-dependent PKB/AKT activity was downregulated. Notably, STAT1-deficient Tregs from tolerized mice failed to protect allografts from rejection *in vivo*. Moreover, STAT1 activation was impaired in alloantigen reactive Tregs isolated from tolerized IFN-γR–deficient as well as IFN-γ–deficient mice. These data suggest that IFN-γ produced by alloantigen reactive Tregs might act on Tregs themselves through an autocrine pathway. Our results propose a previous uncharacterized role of IFN-γ that triggers the STAT1-AKT signaling pathway to control the regulatory function of CD4^+^CD25^+^Foxp3^+^ T cells without affecting Foxp3 expression *in vivo*.

## Materials and Methods

### Mice

129S6/SvEv (129, H2^b^), C57BL/6 (BL6, H2^b^), BALB/c (H2^d^), and 129S6/SvEv Rag2^−/−^ (H2^b^), C57BL/6 Rag1^−/−^ (H2^b^), IFN-γ^−/−^C57BL/6 (H2^b^), IFN-γ Receptor^−/−^ C57BL/6 (H2^b^) mice were bred in the SPF facility, Biomedical Services, John Radcliffe Hospital. STAT1^−/−^129S6/SvEv mice were purchased from Taconic.

### Induction of tolerance, adoptive transfer and skin transplantation

To induce tolerance, adult naïve mice were pretreated with anti-CD4 antibody YTS177 (200 μg i.v days −28 and −27; timing denoted relative to day 0 the time of adoptive transfer) in combination with an infusion of alloantigen (BALB/c (H2^d^) (typically 250 μL whole allogeneic blood i.v. on day −27 and day −1). These were designated tolerized mice. In some experiments, as controls adult mice were treated with an infusion of alloantigen only as above, 1 day before isolation of CD4^+^CD25^+^ T cells. There were designated alloantigen-primed mice. In the adoptive transfer protocol, T-cell–deficient 129S6/SvEv Rag2^−/−^ (H2^b^) or C57BL/6 Rag1^−/−^ (H2^b^) mice were reconstituted i.v. with syngeneic fractionated T cells isolated from tolerized, alloantigen-primed or unmanipulated mice on day 0. The day after reconstitution (day 1), all mice received a BALB/c skin graft as described previously (7). Graft rejection was defined as complete destruction of the skin graft.

### Antibodies and reagents

Fludarabine and PD98059 were purchased from Sigma. Hybridomas YTS 177.9 (anti-CD4) was kindly provided by Professor Herman Waldmann. Mouse CD4^+^ T-cell isolation kits, Golgi Stop reagent, Fix/Perm solution, anti-p-STAT1 (pTyr701), anti-p-PKB/AKT (pSer472/Ser473), anti-PKB/AKT, anti-p-ERK1/2 (pThr202/pTyr204), APC or FITC-labelled anti-CD4 mAb, PE-anti-p-STAT1 (pTyr701), PE-anti-CD25 mAb, PE-Cy7-anti-CD25, anti-CD16/CD32 mAb, FITC-labeled anti-IFN-γ, biotin-anti-IFN-γRα, biotin-anti-IFN-γRβ, PE streptavidin were purchased from BD Biosciences (Oxford, UK). Anti-phospho-STAT5α/β (pTyr694/699) was from Millipore (Upstate) and ECD-anti-CD4 was from Beckman Coulter. Anti-Foxp3 (FJK-16s) and Foxp3 intracellular staining kits were purchased from eBioscience.

### Cell preparation

Single-cell suspensions from mouse spleen were isolated as previously described (16). CD4^+^CD25^+^ and CD4^+^CD25^−^ T cells were purified by flow cytometry using a FACSAria (Becton Dickinson) for *in vivo* experiments. FACS sorted populations were >99% pure.

### Flow cytometric analysis

Standard surface and intracellular cytokine staining were preformed as described earlier (5). Data were acquired using a FACSAria and analyzed using the Diva software package (BD Biosciences).

### Immunoblotting and densitometry

Cell lysates were prepared for SDS-PAGE followed by immunoblotting as previously described earlier (28). ECL films were scanned and the levels of protein phosphorylation or expression were quantified using ImageJ densitometry software. Data are expressed as relative ratios (R) when compared to naïve samples as follows: R = (Dphos/Dtotal)**:**(Dn-phos/Dn-total). Dphos is the density of the phosphorylated protein, Dtotal is the density of the total protein or actin, Dn-phos is the density of the phosphorylated protein from the naïve sample and Dn-total is the density of the total protein or actin from the naïve sample.

### Statistical analysis

Data are presented as mean ± SD. The Student's *t*-test was used to compare two independent groups where the data were normally distributed. Graft survival data analyzed by using the Kaplan–Meier log-rank test were presented as median survival time (MST). Differences were considered to be significant when p < 0.05.

## Results

### The activation of STAT1, but not STAT5 or ERK1/2, is specifically enhanced in alloantigen reactive CD4^+^CD25^+^Foxp3^+^ T cells from alloantigen-tolerized mice

Operational tolerance to alloantigen *in vivo* can be induced in adult mice by pretreatment with donor alloantigen under the cover of anti-CD4 antibody followed by alloantigen specific reactivation 24 h before transplantation (16). We used the same protocol to pre-treat mice and these mice are referred to throughout the paper as ‘tolerized mice’ for clarity (see Materials and Methods). It has been demonstrated that alloantigen reactive CD4^+^CD25^+^ T cells are present in these tolerized mice and that such mice accept donor-type cardiac allografts indefinitely without the need for additional immunosuppression. Therefore, these alloantigen reactive CD4^+^CD25^+^ T cells from tolerized mice are also referred to “tolerized CD4^+^CD25^+^ T cells” throughout for clarity (17,18).

**Figure 1:**
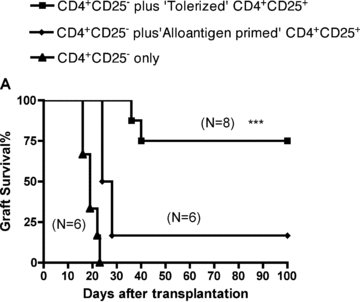

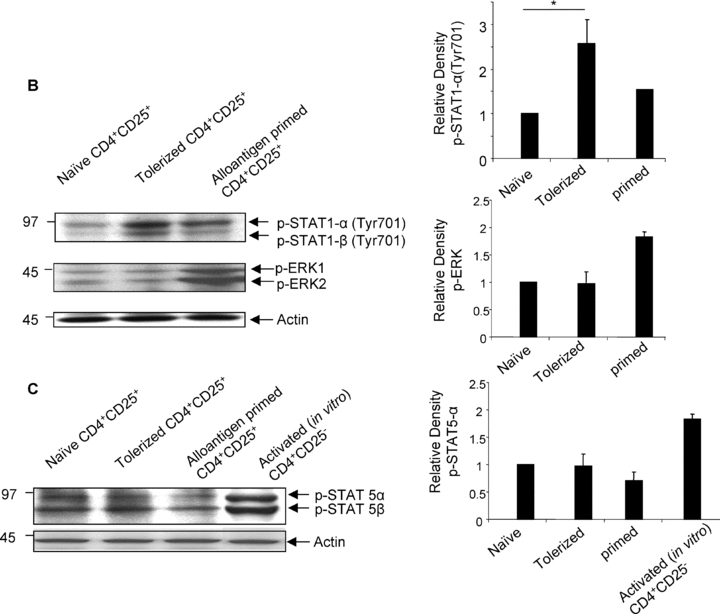

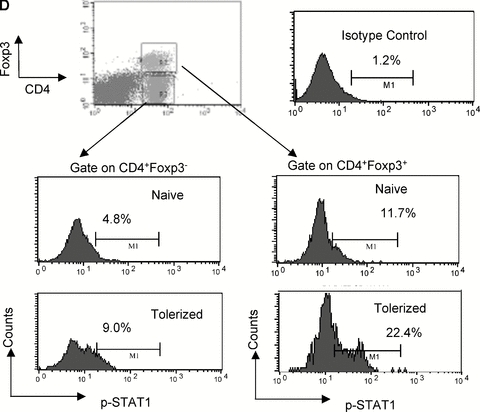
STAT1 phosphorylation is specifically upregulated in CD4^+^CD25^+^Foxp3^+^ T cells from tolerized mice that show an enhanced ability to prevent skin graft rejection. (A) Regulation of skin graft rejection by tolerized CD4^+^CD25^+^ T cells. 129S6/SvEv Rag2^−/−^ mice reconstituted with 2 × 10^5^ CD4^+^CD25^−^ T cells (T_eff_) from 129S6/SvEv mice acutely reject BALB/c skin grafts (n = 6, mean survival time [MST]= 19 days). Cotransfer of 4 × 10^5^ tolerized CD4^+^CD25^+^ T cells (tolerized Tregs) with 2 × 10^5^ syngeneic naïve T_eff_ prevented rejection of BALB/c skin grafts (n = 8, MST > 80 days). In contrast, cotransfer of 4 × 10^5^ alloantigen-primed CD4^+^CD25^+^ cells together with 2 × 10^5^ syngeneic naïve T_eff_ resulted in rejection of BALB/c skin grafts (n = 6, MST = 26 days). BALB/c skin transplants were performed on day 1. Data shown represent three independent experiments. p = 0.0071, tolerized Tregs versus alloantigen primed; p < 0.0001, tolerized Tregs versus T_eff_ only; p = 0.0005, alloantigen-primed CD4^+^CD25^+^ T cells versus T_eff_ only. (B) CD4^+^CD25^+^ T cells purified from tolerized, alloantigen-primed or unmanipulated naïve mice were prepared for SDS-PAGE electrophoresis followed by immunoblotting with anti-phospho-STAT1α/β (Tyr701) (upper panel), anti-phospho-ERK1/2 (pT202/pY204) (middle panel) or anti-actin Ab (lower panel). (C) CD4^+^CD25^+^ T cells from tolerized, alloantigen-primed or unmanipulated naïve mice or CD4^+^CD25^−^ T cells activated with allogeneicirradiated splenocytes together with IL-2 for 48 h *in vitro* were prepared for immunoblotting with anti-p-STAT5α/β mAb (upper panel). Data are expressed as the relative ratios in the right histograms as described in Materials and Methods. The data shown are representative of three independent experiments (*p < 0.05). (D) Splenocytes were isolated from tolerized or unmanipulated C57BL/6 mice and stained with anti-CD4 followed by intracellular anti-Foxp3 and anti-p-STAT1 staining. Events were gated in CD4^+^Foxp3^+^ or CD4^+^Foxp3^−^ populations to assess the levels of p-STAT1 by FACS analysis. FACS profiles are representative of three independent experiments.

We initially found that 4 × 10^5^ tolerized CD4^+^CD25^+^ T cells purified from the spleens of 129S6/SvEv (H2^b^) mice tolerized to BALB/c (H2^d^) alloantigen 24 h after antigen-specific reactivation *in vivo* can prevent the rejection of BALB/c skin grafts initiated by 2 × 10^5^ CD4^+^CD25^−^ T cells from their naïve counterparts (MST >80 days; six of the eight recipients accepted BALB/c skin allografts for >100 days; Figure 1A). However, 4 × 10^5^ recently activated CD4^+^CD25^+^ T cells isolated 24 h after alloantigen infusion into naïve mice (designated ‘alloantigen primed CD4^+^CD25^+^ T cells’) were unable to prevent rejection (i.e. MST = 26 days, five of the six recipients rejected BALB/c skin allografts; Figure 1A). Similar data were obtained in C57BL/6 (H2^b^) mice (data not shown).

To investigate the molecular mechanisms triggered in these tolerized Tregs, STAT1 activation in CD4^+^CD25^+^ T cells purified from tolerized mice was determined by immunoblotting using anti-phospho-STAT1 antibody. The level of phospho-STAT1 was compared to that from alloantigen-primed or naïve CD4^+^CD25^+^ T cells. The active forms of both STAT1α (91KD) and STAT1β (84KD) could be detected by anti-phospho-STAT1 (Tyr701) in CD4^+^CD25^+^ T cells, with STAT1α present at a higher concentration compared with STAT1β (Figure 1B, upper panel). CD4^+^CD25^+^ T cells purified from unmanipulated naïve mice showed a basal level of phosphorylated STAT1α (Figure 1B, upper panel, lane 1). The level of STAT1 phosphorylation in alloantigen-primed CD4^+^CD25^+^ T cells was slightly increased, i.e. 1.4 times higher than that in naive CD4^+^CD25^+^ T cells (Figure 1B, upper panel, lane 3). Interestingly, tolerized CD4^+^CD25^+^ T cells with enhanced regulatory activity significantly increased phosphorylation of both α and β isoforms of STAT1; of the order of two- to three-fold higher than that in naïve CD4^+^CD25^+^ T cells (Figure 1B, upper panel, lane 2) (p < 0.05). Notably, the phosphorylation level of STAT5 was not significantly changed in tolerized CD4^+^CD25^+^ T cells when compared to that in naïve controls (Figure 1C). CD4^+^CD25^−^ T cells activated in the presence of IL-2 *in vitro* were used as a positive control that showed a higher level of STAT5 phosphorylation (Figure 1C). This finding demonstrates that STAT1, but not STAT5 activation is increased in alloantigen reactive CD4^+^CD25^+^ T cells from tolerized mice.

To make sure that tolerized CD4^+^CD25^+^ T cells had regulatory activity and were not recently activated T cells, ERK1/2 activation, a consequent event of T-cell activation, was evaluated by immunoblotting. ERK1/2 phosphorylation levels in tolerized CD4^+^CD25^+^ T cells were comparable to the basal level of ERK1/2 phosphorylation in naïve CD4^+^CD25^+^ T cells, whereas increased ERK1/2 phosphorylation was observed in recently activated alloantigen-primed CD4^+^CD25^+^ T cells (Figure 1B, middle panel and lower histogram).

To further examine whether CD4^+^Foxp3^+^ T cells from tolerized mice could exhibit increased STAT1 phosphorylation levels at the single-cell level, spleen cells from either tolerized C57BL/6 mice or unmanipulated naïve C57BL/6 mice were analyzed for surface CD4 and intracellular Foxp3 and phospho-STAT1 levels by FACS. CD4^+^Foxp3^+^ T cells from the tolerized mice showed approximately a two-fold increase in STAT1 phosphorylation compared to that detected in CD4^+^Foxp3^+^ T cells purified from naive mice (Figure 1D; the percentage of p-STAT1 positive cells was 22.4 ± 0.1% from tolerized mice vs. 11.7 ± 0.9% from naive controls; mean ± SD; n = 3 mice per group). We further confirmed this result using CD4^+^Foxp3/GFP^+^ T cells from tolerized Foxp3gfp.KI mice, which also exhibited enhanced STAT1 phosphorylation levels compared to their naïve or alloantigen-primed counterparts (data not shown).

### Alloantigen reactive STAT1-deficient Tregs fail to control allograft rejection

**Figure 2:**
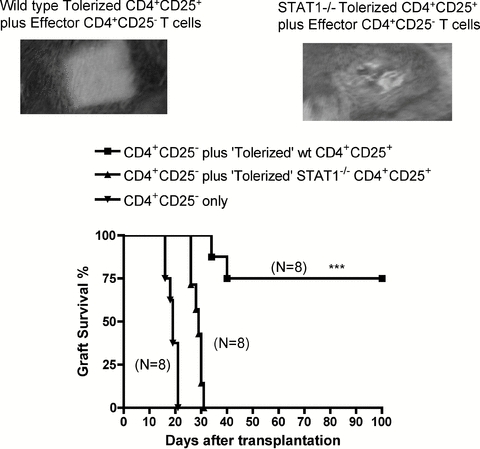
STAT1-deficient CD4^+^ CD25^+^ T cells from tolerized mice fail to prevent skin allograft rejection. Cotransfer of 4 × 10^5^ CD4^+^CD25^+^ T cells from tolerized WT mice (Tolerized Tregs) along with 2 × 10^5^ T_eff_ into 129S6/SvEv Rag2^−/−^ recipients prevents rejection of BALB/c skin grafts (MST = 82 days, n = 8), whereas cotransfer of 4 × 10^5^ CD4^+^CD25^+^ T cells from STAT1^−/−^ tolerized mice and 2 × 10^5^ T_eff_ fails to prevent skin graft rejection (MST = 29.5 days, n = 8, p < 0.0001, tolerized WT Tregs vs. tolerized STAT1^−/−^ Tregs). Upper images are typical accepted transplanted skin (left) and rejected transplanted skin (right). The data shown are representative of three independent experiments.

Because tolerized CD4^+^CD25^+^Foxp3^+^ T cells exhibit the enhanced STAT1 activation (Figure 1), it was important to investigate whether the regulatory activity of tolerized CD4^+^CD25^+^ T cells was dependent on STAT1. 4 × 10^5^ CD4^+^CD25^+^ T cells from either STAT1^−/−^ or wild type (WT) tolerized 129S6/SvEv mice were adoptively transferred into 129S6/SvEv Rag^−/−^ mice together with 2 × 10^5^ CD4^+^CD25^−^ T cells from unmanipulated 129S6/SvEv naïve mice 1 day before a BALB/c skin graft was transplanted. Cotransfer of WT tolerized CD4^+^CD25^+^ T cells along with the CD4^+^CD25^−^ T cells prevented rejection (Figure 2; MST = 82 days). In contrast, cotransfer of STAT1-deficient CD4^+^CD25^+^ T cells from tolerized mice completely abrogated their regulatory capacity (Figure 2; MST = 29.5 days, p < 0.0001). Next, we compared the proliferative response of CD4^+^CD25^−^ T cells from naive mice in the presence of tolerized Tregs that were pretreated with Fludarabine, an inhibitor against STAT1 activity *in vitro* (19). Figure S1 shows that inhibition of STAT1 activity by fludarabine blocks the suppressive function of tolerized CD4^+^CD25^+^Tregs *in vitro*, while inhibition of MAP kinase activity does not affect Tregs function. These observations indicate that STAT1 activation is related to the suppressive function of tolerized Tregs and are consistent with the data obtained using tolerized Tregs isolated from STAT1-deficient mice.

### STAT1 activation in alloantigen-tolerized Tregs is dependent on IFN-γ production

**Figure 3:**
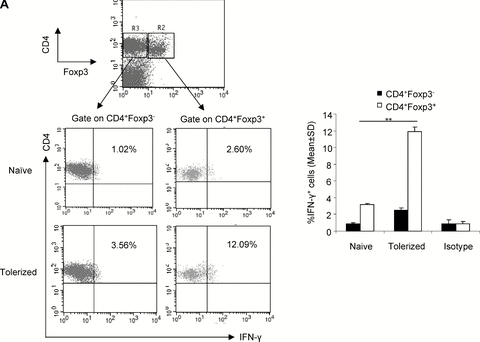

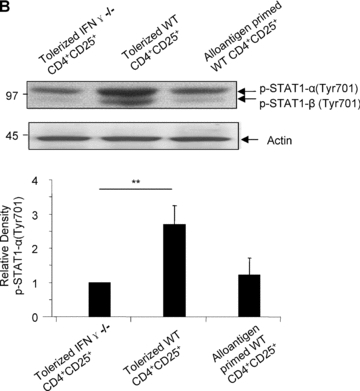
IFN-γ production is upregulated in CD4^+^Foxp3^+^ T cells from tolerized mice. Splenocytes were isolated from tolerized or unmanipulated naïve mice. Surface CD4^+^ together with intracellular Foxp3 and IFN-γ were measured by FACS analysis. The FACS profiles shown are representative of three independent experiments (mean ± SD, n = 3, **p < 0.01). (B) Upregulation of STAT1 phosphorylation in CD4^+^CD25^+^ T cells from tolerized mice is IFN-γ dependent. The phosphorylation levels of STAT1α and β in CD4^+^CD25^+^ T cells from tolerized IFN-γ^−/−^, WT mice or alloantigen-primed WT mice were shown by anti-p-STAT1 immunoblotting (upper panel). Data shown are representative of at least three independent experiments (**p < 0.01).

Next, we measured IFN-γ secretion by tolerized or naïve CD4^+^Foxp3^+^ and CD4^+^Foxp3^−^ T cells at the single-cell level by FACS analysis. A total of 11.9 ± 0.28% of CD4^+^Foxp3^+^ T cells from tolerized mice produced IFN-γ compared to 2.5 ± 0.14% of naïve CD4^+^Foxp3^+^ cells. Further, it was noted that CD4^+^Foxp3^+^ T cells greatly increased IFN-γ production compared to CD4^+^Foxp3^−^ T cells in tolerized mice (11.9 ± 0.28% vs. 3.2 ± 0.56%; n = 3 mice per group; Figure 3A) and that this phenotype was also seen in naïve mice but to a lesser degree (2.5 ± 0.14% vs. 1.02 ± 0.11%). These data demonstrate that tolerized CD4^+^Foxp3^+^ T cells produce significantly higher levels of IFN-γ than naïve CD4^+^Foxp3^+^ T cells.

In addition to IFN-γ, other stimuli including IFN-α and platelet-derived growth factor also affect STAT1 phosphorylation (20,21). We next determined whether the increased STAT1 phosphorylation observed in Tregs from tolerized mice was caused by IFN-γ*in vivo.* STAT1 activation was assessed in CD4^+^CD25^+^ T cells isolated from IFN-γ–deficient versus WT tolerized mice (Figure 3B). The level of STAT1 phosphorylation in IFN-γ–deficient tolerized CD4^+^CD25^+^ T cells was markedly reduced compared to that observed in WT tolerized CD4^+^CD25^+^ T cells (Figure 3B, upper panel, lane 1 vs. lane 2; and lower histogram). Our previous findings demonstrated that Tregs generated in IFN-γ–deficient mice have impaired suppressive function and ability to control skin graft rejection (adoptive transfer of naïve T_eff_ and IFN-γ^−/−^ Tregs resulted in seven out of nine mice rejecting their skin grafts) (7). Together, these data indicate that IFN-γ produced by Tregs might enhance STAT1 activation thereby modifying Tregs function.

### Enhanced STAT1 activation in tolerized CD4^+^CD25^+^ T cells is dependent on IFN-γ receptor ligation

**Figure 4:**
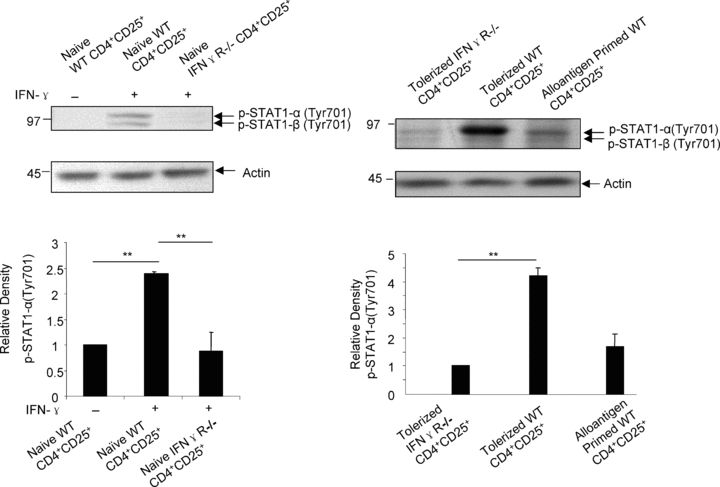
STAT1 phosphorylation is dependent on IFN-γ receptor. (A) Naïve CD4^+^CD25^+^ T cells respond to IFN-γ via their IFN-γR. CD4^+^CD25^+^ T cells from naïve WT or IFN-γR^−/−^ mice were treated with or without exogenous IFN-γ (2 U/μL) for 20 min, followed by immunoblotting with anti-p-STAT1α and β (upper panel). (B) Upregulation of STAT1 phosphorylation in Tregs from tolerized mice is IFN-γ receptor dependent. STAT1α phosphorylation levels in CD4^+^CD25^+^ T cells purified from either tolerized IFN-γR^−/−^ or WT mice or alloantigen-primed WT mice were shown by anti-phospho-STAT1 blotting (upper panel). Data shown are representative of three independent experiments (*p < 0.05, **p < 0.01).

To ask whether the IFN-γR expressed by CD4^+^CD25^+^ T cells in tolerized mice was responsible for IFN-γ–induced STAT1 phosphorylation, we first confirmed that IFN-γRs was expressed on CD4^+^CD25^+^ T cells (Figure S2). Exogenous IFN-γ treatment significantly increased the level of STAT1α phosphorylation in WT CD4^+^CD25^+^ T cells, which was 2.7-fold higher than the basal level in naïve untreated CD4^+^CD25^+^ T cells (Figure 4A, lane 2 vs. lane 1, p < 0.01). In contrast, STAT1α phosphorylation remained at the basal level in CD4^+^CD25^+^ T cells from IFN-γR^−/−^ mice after exogenous IFN-γ stimulation (Figure 4A, upper panel, lane 3). Next, we investigated whether enhanced intrinsic IFN-γ production by tolerized CD4^+^CD25^+^ T cells could directly ligate IFN-γRs on the CD4^+^CD25^+^ T cells themselves and thus modulate STAT1 activity. Approximately four-fold higher levels of STAT1α phosphorylation were observed in WT tolerized CD4^+^CD25^+^ T cells than in IFN-γR^−/−^ CD4^+^CD25^+^ T cells (Figure 4B; p < 0.01; n = 3). It is worth noting that the STAT1α phosphorylation level in IFN-γR^−/−^ tolerized CD4^+^CD25^+^ T cells was even lower than that detected in alloantigen-primed WT mice (Figure 4B, upper panel, lane 1 vs. lane 2). We also observed that IFN-γR—deficient Tregs had a reduced suppressive ability *in vivo* (four out of six mice reconstituted with naïve T_eff_ and IFN-γR^−/−^ Tregs rejected allogeneic skin grafts, data not shown). These data suggest that IFN-γR is important for STAT1 activation in tolerized Tregs and that increased IFN-γ production in tolerized Tregs might ligate IFN-γ receptors on themselves to induce the intracellular JAK-STAT1 signaling pathway in an autocrine manner.

Comparable levels of Foxp3 expression were detected in CD4^+^CD25^+^ T cells from unmanipulated naïve, alloantigen-primed and tolerized mice (Figure S3A). The percentages of Foxp3 positive cells in CD4^+^CD25^+^ T cells from unmanipulated naïve, alloantigen-primed and tolerized mice were also comparable (data not shown). Importantly, expression of Foxp3 by CD4^+^CD25^+^ cells purified from STAT1^−/−^ or IFN-γR^−/−^ mice was equivalent to that from WT Tregs (Figure S3B). These data indicate that IFN-γ–STAT1 signaling plays a key role in the regulatory function of alloantigen-tolerized Tregs *in vivo* without affecting Foxp3 expression.

### The down-regulated PKB/AKT phosphorylation in tolerized CD4^+^CD25^+^ Tregs is STAT1 dependent

**Figure 5:**
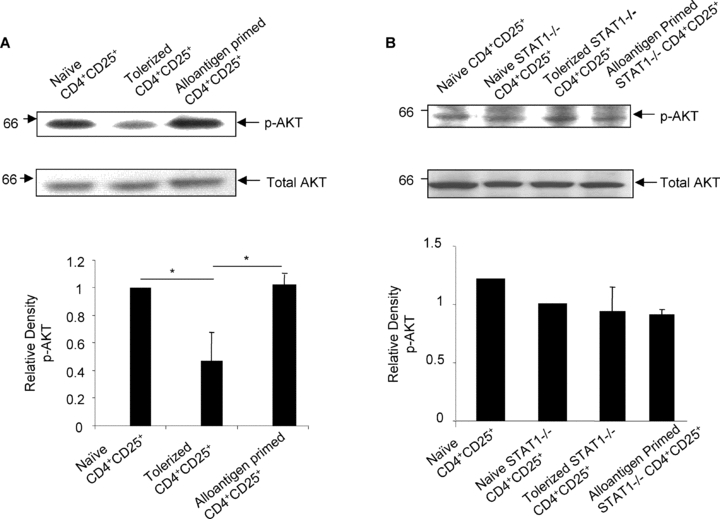

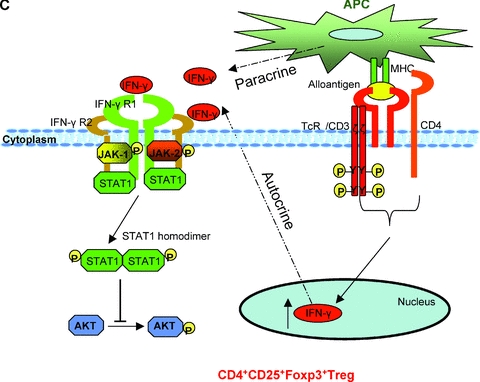
STAT1-dependent PKB/AKT phosphorylation is down-regulated in tolerized CD4^+^CD25^+^ Tregs *in vivo*. (A) Lysates from tolerized CD4^+^CD25^+^ T cells, alloantigen-primed CD4^+^CD25^+^ or naïve CD4^+^CD25^+^ T cells were prepared for immunoblotting with anti-phospho-PKB/AKT (upper panel) and anti-PKB/AKT1/2 Ab (lower panel) (*p < 0.05). (B) Naïve WT, naïve STAT1^−/−^ CD4^+^CD25^+^, tolerized STAT1^−/−^ CD4^+^CD25^+^ or alloantigen-primed STAT1^−/−^ CD4^+^CD25^+^ were prepared for immonoblotting with anti-phospho-PKB/AKT Ab (upper panel) and anti-PKB/AKT1/2 Ab (lower panel). Data shown are representative of at least three independent experiments. (C) Model: IFN-γ triggered JAK-STAT1-PKB/AKT signalling pathway controls the function of CD4^+^CD25^+^Foxp3^+^ regulatory T cells *in vivo* in an autocrine/paracrine manner. Tolerized CD4^+^CD25^+^Foxp3^+^ Tregs adapted by alloantigen and anti-CD4 can upregulate IFN-γ secretion. The IFN-γ released by Tregs in the local microenvironment might ligate the IFN-γ receptor on Tregs themselves to induce the JAK-STAT1 signalling pathway in an autocrine manner. APCs could also provide IFN-γ that might upregulate the JAK-STAT1 pathway in Tregs in a paracrine manner. Furthermore, IFN-γ triggers STAT1-dependent downregulation of PKB/AKT activation in Tregs. The up-regulated STAT1 activation and down-regulated PKB/AKT activation regulates the suppressive function of Tregs to protect the graft *in vivo*.

A link between JAK-STAT1 activation and PKB/AKT activation has been reported in erythroblasts in response to erythropoietin (22). However, it is not known whether IFN-γ–induced JAK-STAT1 activation controls PKB/AKT activation in CD4^+^CD25^+^ Tregs. To answer this question, the phosphorylation level of PKB/AKT was first measured in tolerized CD4^+^CD25^+^ T cells by immunoblotting with anti-phospho-PKB/AKT (S472/473) mAb. Compared to those from unmanipulated naïve mice, the phosphorylation level of PKB/AKT was significantly decreased in tolerized CD4^+^CD25^+^ T cells (i.e. only R = 0.47 ± 0.22; p < 0.05; Figure 5A). Recently activated CD4^+^CD25^+^ T cells from alloantigen-primed mice showed a comparable level of phospho-AKT compared to naïve CD4^+^CD25^+^ T cells (R = 1.05 ± 0.11; Figure 5A). Next, it was important to address whether downregulation of PKB/AKT activation in tolerized CD4^+^CD25^+^ T cells was STAT1 dependent. Interestingly, the level of phospho-AKT was restored in CD4^+^CD25^+^ T cells from STAT1-deficient tolerized mice, such that it was comparable to those from either naïve WT mice or naïve/alloantigen-primed STAT1-deficient mice (Figure 5B).

These data together indicate that tolerized Tregs upregulate IFN-γ production, which enhances STAT1 activation, but suppresses STAT1-dependent AKT activation. This signaling pathway is important for the capacity of tolerized Tregs to prevent allogeneic skin graft rejection *in vivo*.

## Discussion

Here we demonstrate that enhanced IFN-γ produced by CD4^+^CD25^+^Foxp3^+^ Tregs from mice tolerized to alloantigen *in vivo* can ligate IFN-γ receptors on the Tregs themselves to upregulate STAT1 activation and decrease PKB/AKT activation (Figures 1, 3, 4 and 5). By contrast, STAT5 or ERK phosphorylation/activation is not affected (Figure 1B, C). Importantly, the enhanced STAT1 activation and decreased AKT activation are in the same signaling pathway induced by IFN-γ in Tregs (Figure 5B), and is required for alloantigen reactive Tregs from tolerized mice to control allogeneic skin graft rejection *in vivo* (Figure 2).

It was interesting to note that CD4^+^Foxp3^+^ Tregs showed significantly increased STAT1 phosphorylation compared to CD4^+^Foxp3^−^ T cells from either unmanipulated naïve mice or tolerized mice (Figure 1D and Supporting Figure S1). This might indicate that compared to CD4^+^Foxp3^−^ cells in the same microenvironment, CD4^+^Foxp3^+^ Tregs can lower the threshold to activate STAT1 in response to the local production of IFN-γ*in vivo* by Tregs themselves or by other cell types. In addition, it was noted that alloantigen reactive CD4^+^Foxp3^+^ Tregs further increase IFN-γ production compared to naïve Tregs (Figure 3A). This might be one of the critical sources of IFN-γ within the microenvironments, that is the graft and the draining lymphoid tissue (23) where alloantigen reactive Tregs respond to IFN-γ and enhance STAT1 activity *in vivo*. Importantly, we found that STAT1 deficiency impaired the suppressive function of tolerized Tregs *in vivo* (Figure 2). Others have also demonstrated that STAT1-deficient mice have an increased susceptibility to autoimmune disease that is linked to the presence of a reduced number of Tregs (10).

We recently showed that in the early phase of the response after transplantation, Tregs are resident in the draining lymphoid tissue where they can prevent the priming of effector T cells. Later in the response, Tregs migrate to the graft where they control effector T-cell populations to prevent the development of memory T cells (23). It is possible that these alloantigen-specific Tregs from tolerized mice might be preferentially recruited to the graft site *in vivo,* since the grafted skin provides alloantigen that can be presented by local antigen-presenting cells (APCs) to attract these alloantigen reactive Tregs. Moreover, local multiple stimuli such as IFN-γ might further enhance STAT1 activity in Tregs to maintain their presence within the graft and to enhance their regulatory role. Consistent with this hypothesis, it has been suggested that IFN-γ inhibits monocyte migration and traps these cells at inflammation sites through the downstream STAT1 (24). It would be interesting to further investigate whether IFN-γ–triggered JAK-STAT1 signals plays an important role in the recruitment and maintenance of Foxp3^+^ T cells at inflammatory sites, such as a graft.

Another important observation is that STAT1 activation was abrogated in CD4^+^CD25^+^ Tregs from tolerized IFN-γ–deficient mice (Figure 3B) as well as from IFN-γR–deficient mice (Figure 4B). These data suggest a model that when CD4^+^CD25^+^Foxp3^+^ T cells in tolerized mice reencounter alloantigen they upregulate IFN-γ production into the local microenvironment, such that where IFN-γ ligates IFN-γ receptors on the Tregs themselves it induces JAK-STAT1 signaling through an autocrine loop (Figure 5C), thus enhancing their regulatory function to protect the graft. This observation is consistent with findings from other laboratories, demonstrating that IFN-γ exerts its effects on cells by interacting with its specific receptors (8). The source of IFN-γ may not only come from the Tregs themselves but also from other cell types, including recently activated Th1 cells and NK cells and APCs (8). Further, IFN-γ also affects the function of other cell types, such as the expression of indoleamine-2,3-dioxygenase (IDO) by macrophages or dendritic cells (DCs). IDO-induced tryptophan breakdown is necessary for maintenance of various aspects of immune tolerance (25). Interestingly, IFN-γR–deficient APCs abrogated IDO expression when exposed to alloantigen reactive Tregs and this resulted in the rejection of transplanted skin (B. Wei et al., unpublished data). These findings support a model whereby IFN-γ produced by tolerized Tregs might not only directly ligate IFN-γ receptors to induce JAK-STAT1 signaling to influence the function of the Tregs themselves, but also induce IDO expression in APCs to indirectly regulate tolerance induction (Figure 5C).

IFN-γ has been reported to display inhibitory activity against Th17 cells that have been reported to play a pathogenic role in type I diabetes and cause inflammation (26). Although Th17 cell development does not require STAT1 signaling (27), it is likely that the enhanced IFN-γ production by alloantigen reactive Tregs might inhibit the function of the Th17 lineage while favoring the function of Tregs in the graft. These combined influences might enhance the overall protective effects of Tregs promoting graft survival.

In summary, our study demonstrates that the enhanced IFN-γ production by alloantigen reactive Tregs from tolerized mice can ligate IFN-γR on Tregs cells themselves to upregulate STAT1 activation and downregulate PKB/AKT activation. Further, the IFN-γ triggered STAT1-PKB/AKT signaling pathway increases the capacity of alloantigen reactive Tregs to prevent allograft rejection *in vivo.* This study also provides a molecular mechanism to support the functional data previously reported in models of autoimmunity (6) and transplantation (7). It is important to understand if these observations in mice have relevance to the function of human Tregs. Our preliminary data show that human CD4^+^CD25^hi^ Tregs also have the capacity to secrete IFN-γ upon restimulation (Figure S4). We are currently investigating whether IFN-γ plays a role in the function of human Tregs as this may help us to understand whether it is possible to target IFN-γ–induced JAK-STAT1-AKT signaling pathways to prolong allograft survival, prevent graft-versus-host disease or treat autoimmune diseases.

## Supporting Information

**Figure S1. CD4^+^CD25^+^ T cells with STAT1 inhibition fail to suppress T-cell proliferation.** Purified CD4^+^CD25^−^T cells (1 × 10^5^ cells/well) were cultured with UV-irradiated TDS (T-cell-depleted splenocytes, 5 × 10^5^ cells/well) in a 96-well round-bottom plate. For stimulation, 1 μg/mL anti-CD3 antibody was added. To analyze the suppressive properties of Tregs, syngeneic CD4^+^CD25^+^ T cells were pretreated with STAT1 inhibitor Fludarabine (F, 25 μM), MAPK inhibitor PD98059 (PD, 10 μM) or media control, and the indicated ratios of CD4^+^CD25^+^ Treg cells (between CD4^+^CD25^−^ T cells and Treg cells) were added into the 96-well round-bottom plate. After 3 days of incubation, proliferation of responder T cells was determined by [^3^H] thymidine incorporation (1 μCi/well) for the last 16 h of culture. These results are representative of three independent experiments (*p < 0.05, **p < 0.01).

**Figure S2. Surface expression levels of IFN-γR in CD4^+^CD25^+^ cells were measured by FACS as described in Materials and Methods.**

**Figure S3. (A) Foxp3 expression levels are normal in CD4^+^CD25^+^ T cells from tolerized mice or alloantigenprimed mice.** CD4^+^CD25^+^ T cells or CD4^+^CD25^−^ T cells were isolated from tolerized mice, alloantigen-primed or naïve unmanipulated mice, and prepared for immunoblotting with anti-Foxp3 mAb (upper panel) and anti-actin mAb (lower panel). (B) IFN-γ triggered STAT1 activation has no effect on Foxp3 expression in CD4^+^CD25^+^ T cells. Splenocytes from WT, STAT1^−/−^, IFN-γR^−/−^ mice were recovered and analyzed for CD4, CD25 and Foxp3 expression by FACS. Data represent the percentage of Foxp3^+^ T cells (left) or MFI (mean fluorescence intensity) of Foxp3 staining in CD4^+^CD25^+^ populations (right). The data shown are representative of three independent experiments.

**Figure S4. Human CD4^+^CD25^hi^ Foxp3^+^ Tregs can produce limited amounts of IFN-γ.** Human CD4^+^CD25^high^ T cells with regulatory activity were sorted and expanded *in vitro* with anti-CD3/anti-CD28 coated beads (Miltenyi Biotech) in the presence of IL-2. The cells were then stimulated with 100 ng/mL phorbol 12-myristate 13-acetate (PMA), 1 γM ionomycin and 2 γl/mL Golgi Stop for 5 h. Recovered cellswere stained with ECD-anti-CD4 and PE-Cγ7-anti-CD25 before fixing with Fix/Perm solution for 45 min, then washed with permeabilization buffer and incubated with 2% rat serum for 15min. Next, cellswere stained with FITC-anti-FoxP3 and PE-anti-IFN-γ antibody for 30 min. The data shown are representative of three independent experiments.
